# New Insights on the Adjuvant Properties of the *Leishmania infantum* Eukaryotic Initiation Factor

**DOI:** 10.1155/2019/9124326

**Published:** 2019-04-30

**Authors:** Olga S. Koutsoni, Mourad Barhoumi, Ikram Guizani, Eleni Dotsika

**Affiliations:** ^1^Laboratory of Cellular Immunology, Department of Microbiology, Hellenic Pasteur Institute, 127 Vass Sofias Av, 11521 Athens, Greece; ^2^Department of Microbiology, Faculty of Medicine, National and Kapodistrian University of Athens, Athens, Greece; ^3^Laboratory of Molecular Epidemiology and Experimental Pathology, Institut Pasteur de Tunis/Université de Tunis el Manar, 13 Place Pasteur, BP 74, 1002 Tunis-Belvédère, Tunisia

## Abstract

Vaccination is the most effective tool against infectious diseases. Subunit vaccines are safer compared to live-attenuated vaccines but are less immunogenic and need to be delivered with an adjuvant. Adjuvants are essential for enhancing vaccine potency by improving humoral and cell-mediated immune responses. Only a limited number of adjuvants are licensed for human vaccines, and their mode of action is still not clear. *Leishmania* eukaryotic initiation factor (LeIF) has been described having a dual role, as a natural adjuvant and as an antigen that possesses advantageous immunomodulatory properties. In this study, we assessed the adjuvant properties of recombinant *Leishmania infantum* eukaryotic initiation factor (LieIF) through *in vitro* and *in vivo* assays. LieIF was intraperitoneally administered in combination with the protein antigen ovalbumin (OVA), and the widely used alum was used as a reference adjuvant. Our *in vitro* studies using J774A.1 macrophages showed that LieIF induced stimulatory effects as demonstrated by the enhanced surface expression of CD80 and CD86 co-stimulatory molecules and the induced production of the immune mediators NO and MIP-1*α*. Additionally, LieIF co-administration with OVA in an *in vivo* murine model induced a proinflammatory environment as demonstrated by the elevated expression of *TNF-α*, *IL-1β*, and *NF-κB2* genes in peritoneal exudate cells (PEC). Furthermore, PEC derived from OVA-LieIF-immunized mice exhibited elevated expression of CD80 molecule and production of NO and MIP-1*α* in culture supernatants. Moreover, LieIF administration in the peritoneum of mice resulted in the recruitment of neutrophils and monocytes at 24 h post-injection. Also, we showed that this immunopotentiating effect of LieIF did not depend on the induction of uric acid danger signal. These findings suggest the potential use of LieIF as adjuvant in new vaccine formulations against different infectious diseases.

## 1. Introduction

Vaccines are an indisputable achievement of medical science since millions of lives have been saved from infectious diseases, while they also contribute significantly in reducing healthcare expenditure [1]. Nowadays, there are still several diseases that cause significant morbidity and mortality worldwide because either there is no access to vaccine market or the existing vaccines confer suboptimal protection. Another factor is the emergence of new pathogens or re-emergence of old ones [2]. New technologies divided into three major categories related to antigen discovery, adjuvants and vaccine vector delivery and deciphering human immune responses, have recently been developed providing a revolution in vaccine development [3].

The term adjuvant, derived from the Latin word adjuvare that means “to help” [4], comprises all compounds that have the ability to enhance and/or shape antigen-specific immune responses [5, 6]. Adjuvants are used in vaccine formulations in order to enhance the immunogenicity of highly purified native or recombinant antigens, to reduce the amount of antigen or the number of immunizations needed for the establishment of a protective immunity, and generally to improve the efficacy of vaccine formulations. Therefore, identification and determination of mode of action of potent adjuvants are particularly important for vaccine discovery [7].

Vaccine adjuvants represent a diverse class of compounds, such as microbial products (e.g., pertussis toxin, cholera toxin, bacterial flagellin, and heat shock proteins), cytokines (e.g., IL-12, IFN-*γ*, and granulocyte-macrophage colony-stimulating factor (GM-CSF)), toll-like receptor agonists (e.g., LPS, poly(I:C), and CpG), mineral salts (e.g., alum), emulsions (e.g., MF59 and Freund's), microparticles, liposomes, and virosomes [8, 9]. So far, very few adjuvants are being used in licensed human and livestock vaccines, such as alum, MF59, monophosphoryl lipid A plus alum (AS04), and saponin (QS-21) [8, 10].

Various adjuvants exert their functions through different mechanisms of action including formation of antigen depot, induction of immune mediators such as cytokines and chemokines, activation of antigen-presenting cells (APCs) (e.g., dendritic cells (DCs)), enhancement of antigen uptake by APCs, and induction of local inflammation and cellular recruitment [11]. Delineation of adjuvants' mode of action provides valuable scientific knowledge for the induction of competent interplay among innate and adaptive immunity, while the deep understanding of the mechanism of action of adjuvants is indispensably important in expediting their development.

Until now, alum-based compounds still remain the predominant human adjuvants due to their safety, ease of preparation, and stability [2]. Thus, alum is found in numerous commercial vaccines including HAV, HBV, HPV, diphtheria and tetanus (DT), Haemophilus influenzae type B (HIB), and pneumococcal conjugate vaccines [12]. Alum's mechanism of action includes the depot effect even though there are reports demonstrating that depot formation is not required for alum adjuvanticity [13, 14], the induction of Th2-type immune responses, the stimulation of inflammation at the injection site like the production of proinflammatory cytokines, and the recruitment of innate immune cells [15, 16]. However, the use of alum presents several drawbacks: (a) is a poor inducer of T-cell mediated responses in humans, namely, Th1-type or cytotoxic T-cell responses which are essential in protective immunity against intracellular pathogens (such as *Leishmania*) and (b) vaccines containing alum cannot be sterilized by standard methods, e.g., filtration, be deep frozen, or be lyophilized [2]. Thus, the development of new effective vaccine formulations that require strong cellular-mediated immunity needs the use of appropriate adjuvants.

Leishmaniasis is a tropical and subtropical disease found in 98 countries, while the achievement of developing safe, effective, durable, and low-cost prophylactic vaccines against the disease is still a major challenge [17]. Several native and recombinant *Leishmania* proteins have been successfully tested as vaccine candidate antigens against leishmaniasis revealing a number of important immune compounds that determine the immune outcome towards protection or exacerbation of experimental infections [18]. Interestingly, among these *Leishmania* proteins, recombinant *Leishmania* eukaryotic initiation factor (LeIF) has been described as an antigen able to induce a protective Th1-type immune response against leishmaniasis [19, 20]. LeIF protein has 403 residues and is highly conserved among *Leishmania* species, also showing high sequence similarity to the mammalian translation initiation factor eIF4A [20, 21]. It has also advantageous immunomodulatory properties, like induction of the production of Th1-type cytokines, IL-12 and IFN-*γ*, by human peripheral mononuclear cells (PBMCs) from either leishmaniasis patients or normal individuals [19]. It is also able to induce the production of IL-12, IL-10, and TNF-*α* by monocytes, macrophages, and DCs derived from healthy volunteers [20, 22, 23]. Additionally, we have recently shown that recombinant *Leishmania infantum* eukaryotic initiation factor (LieIF) in the presence of IFN-*γ* inhibits *L. donovani* growth in murine macrophages [24] and is able to induce phenotypic maturation and functional differentiation of murine bone marrow-derived DCs (unpublished data). Moreover, the NH_2_-terminal part (1-226) of LeIF, known to preserve its immunomodulatory properties [19, 20], has been incorporated in a trifusion recombinant protein vaccine, Leish-111f, which was shown to be protective in mice models, when administered in association with immune adjuvants [25–27]. Furthermore, the Leish-111f protein vaccine formulated with the monophosphoryl lipid A (MPL) adjuvant in an oil-in-water stable emulsion using synthetic squalene (MPL-SE) has been tested in clinical trials demonstrating its safety and immunogenicity, supporting the future plan for its clinical development in prophylaxis of human cutaneous and mucosal leishmaniasis (ClinicalTrials.gov Identifier: NCT00121862, NCT00121849, NCT00111553, NCT00111514, and NCT00486382) [28]. In addition, LeIF has been used as adjuvant to promote the induction of Th1-type immune response against the tumor-associated MUC1 tandem repeat peptide in a chimpanzee animal model [29]. It has been shown that the vaccination with tumor-associated MUC1 tandem repeat peptide in combination with LeIF induced proliferative T cell responses and expression of IFN-*γ* by CD4+ peripheral blood and lymph node T cells in immunized chimpanzees [29].

Until recently, adjuvant selection was empirical and despite the wide use of alum adjuvant in licensed human vaccines, their mode of action is not well characterized. In the present study, we present data showing the potential of LieIF to provide adjuvant properties in *in vitro* and *in vivo* assays. To achieve this objective, recombinant LieIF adjuvant was tested *in vitro* for its ability to potentiate antigen presentation properties of J774A.1 macrophages and *in vivo* for its capability to generate the requisite cellular environment favoring the development of adaptive immune responses.

## 2. Materials and Methods

### 2.1. Laboratory Animals

Six- to eight-week-old female BALB/c mice were obtained from the breeding unit of Hellenic Pasteur Institute (HPI; Athens, Greece). All experimental animals were housed in a specific pathogen-free animal facility, at a temperature of 22-25°C and a photoperiod of 12 h. They received a balanced diet of commercial food pellets and water *ad libitum*. The reporting of the animal experiments in this study followed the ARRIVE guidelines. *In vivo* experimentation was approved by the Institutional Protocols Evaluation Committee according to PD 56/2013 as adoption of Directive 2010/63/EU. Protocol license was issued by the Official Veterinary Authorities of the Prefecture of Attiki in compliance with the above legislation in force.

### 2.2. Macrophage Culture

The immortalized J774A.1 macrophage cell line was purchased from the American Type Culture Collection (ATCC; Rockville, USA/ATCC No. TIB-67). The J774A.1 macrophage cells were cultured in complete RPMI-1640 medium (Biochrom AG, Berlin, Germany), i.e., RPMI-1640 supplemented with 2 mM L-glutamine, 10 mM Hepes, 24 mM NaHCO_3_, 50 *μ*M of 2-mercaptoethanol, 100 U/mL penicillin, 100 *μ*g/mL streptomycin, and 10% *v*/*v* heat-inactivated fetal bovine serum (FBS; Gibco, Paisley, UK). Cells were maintained in 25 cm^2^ cell culture flasks (CELLSTAR, Greiner Bio-one, Germany), at 37°C with 5% CO_2_ environment. J774A.1 macrophage cells were cultured to 80% confluence, and monolayers of cells were routinely harvested by gentle scraping with a cell scraper and diluted 1 : 5 in fresh medium. Cells were counted in a Malassez hemocytometer, and the viability (>95%) of J774A.1 cells was determined by trypan blue exclusion dye.

### 2.3. Cloning, Expression, and Purification of LieIF Protein

**Figure 1:**
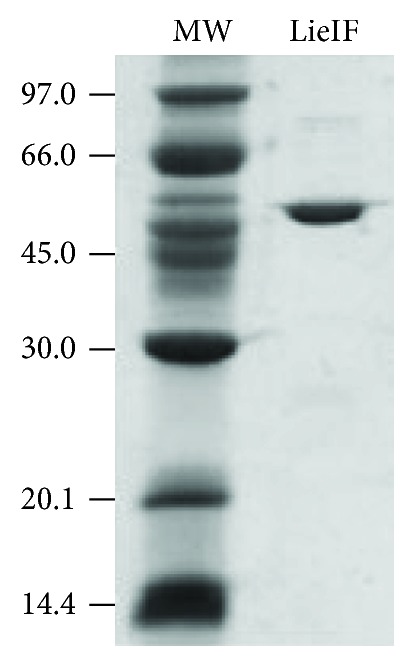
Expression and purification of the recombinant LieIF protein. Aliquots of purified protein were resolved by SDS-PAGE gel and stained with Coomassie Brilliant Blue. The positions of the Bio-Rad prestained markers (in kDa) are indicated at the left.

The *LieIF* gene was amplified from *L. infantum* (MHOM/TN/88/Aymen) genomic DNA by PCR, as previously described [24]. The LieIF construct was subcloned into the *NdeI* and *XhoI* sites of pET-22b expression vector (Novagen, San Diego, CA, USA). LieIF protein was expressed in Origami (DE3) *E. coli* strain (Novagen) and purified using Ni-affinity chromatography, as previously reported [22]. Protein concentration was determined using the Bio-Rad Protein Assay (Hercules, CA, USA) with the use of bovine serum albumin (BSA) as a standard while its purity was verified on a 12% Coomassie-stained SDS-PAGE gel (Figure 1). Recombinant LieIF was tested for the amount of endotoxin levels (≤5 EU/mg) using the *Limulus* amebocyte lysate (LAL) assay (Charles River, USA).

### 2.4. Antigens and Adjuvant

OVA antigen was purchased from Sigma-Aldrich Corp. (USA) and was also tested for bacterial endotoxin using the LAL assay. At the dose used in our experiments, the endotoxin level of OVA was ≤0.001 *μ*g/mL. Imject Alum adjuvant (Pierce, Rockford, USA) is a mixture of aluminum hydroxide and magnesium hydroxide and was mixed at a 1 : 1 ratio with a solution of OVA antigen in phosphate-buffered saline (PBS) pH = 7.4, followed by stirring for at least 1 h to effectively absorb the antigen.

### 2.5. Immunization Protocols

**Figure 2:**
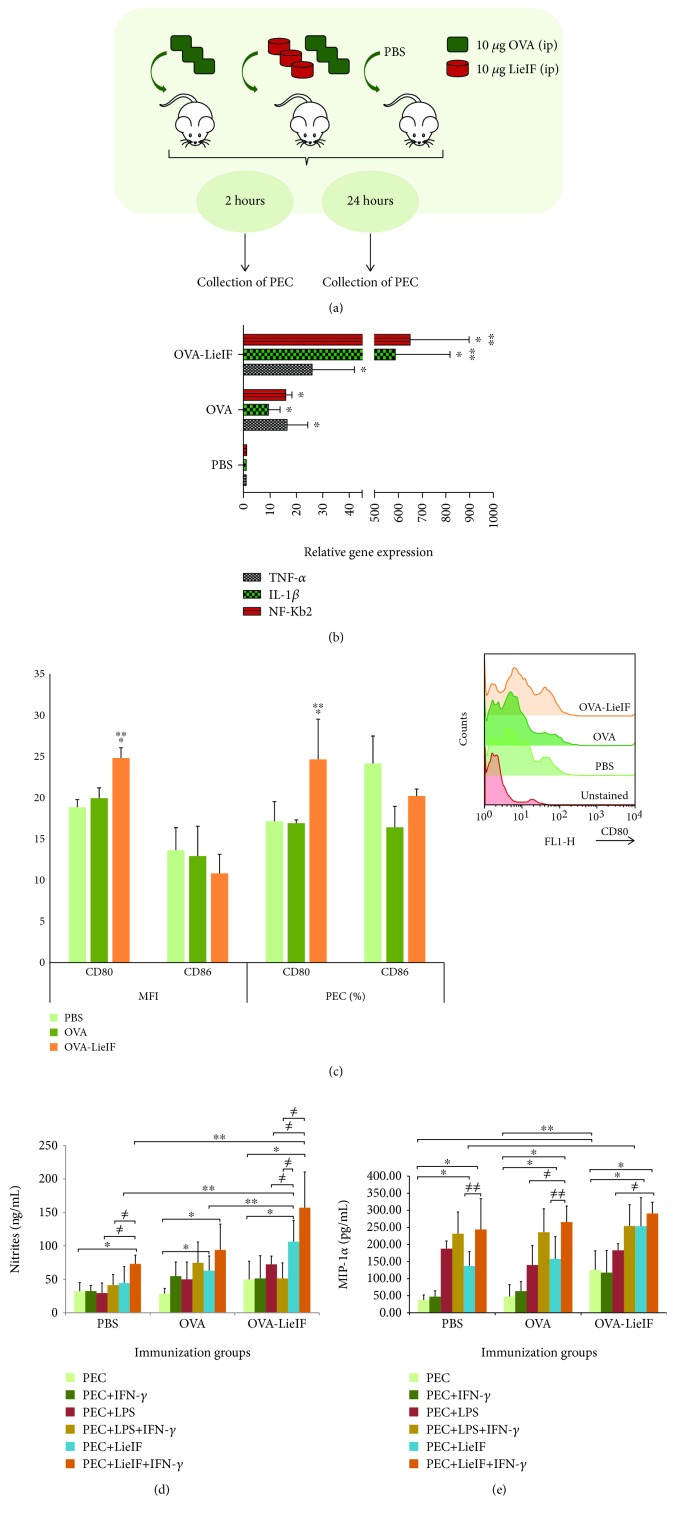
*In vivo* effect of recombinant LieIF protein on the peritoneal exudate cells. (a) Schematic representation of the experimental protocol. Female BALB/c mice were i.p. injected in the right quadrant with 500 *μ*L of LieIF suspension (10 *μ*g/mouse) in sterile PBS containing equal quantity of OVA protein (10 *μ*g/mouse) or with 500 *μ*L of OVA suspension (10 *μ*g/mouse) in sterile PBS, while mice receiving only PBS were included. 2 and 24 h after injection, the peritoneal exudate cells (PEC) were harvested with 5 mL of ice-cold PBS. (b) Relative expression of *TNF-α*, *IL-1β*, and *NF-κB2* genes in PEC. 2 h post-immunization, PEC were derived from each experimental group and relative expression of *TNF-α*, *IL-1β*, and *NF-κB2* genes was determined by real-time PCR, performed with a SYBR Green PCR Master Mix. The expression of glyceraldehyde-3-phosphate dehydrogenase (GAPDH) gene was used for normalization, and all expression levels were computed via the ΔΔCt method. Results shown are representative of three independent experiments. ∗ indicates statistically significant differences compared to PBS-immunized mice while ∗∗ indicates significant differences compared to OVA-immunized group. (c) Recombinant LieIF protein induces the upregulated expression of CD80 co-stimulatory molecule in PEC. 24 h post-immunization, PEC were harvested from each experimental group and cell surface expression of CD80 and CD86 co-stimulatory molecules was assessed by FACS analysis. The results are expressed as median fluorescent intensity (MFI) and as percentage (%) of cells expressing CD80 and CD86 molecules. Data are presented as mean values ± SD of three independent experiments. The histogram overlay is representative of one experiment. ∗ and ∗∗ indicate statistically significant differences as compared to PBS- and OVA-immunized groups, respectively. (d) Recombinant LieIF protein promotes the production of NO by PEC. 24 h post-immunization, PEC were harvested from each experimental group and were further incubated *in vitro* with LieIF (10 *μ*g/mL), IFN-*γ* (1 ng/mL), and LPS (1 *μ*g/mL) or with LieIF+IFN-*γ* and LPS+IFN-*γ*, for 24 h at 37°C under 5% CO_2_ environment. After the incubation period, NO production of each experimental group was determined in supernatants with the Griess reaction. Data are presented as mean values ± SD of three independent experiments. For each *in vivo* experimental group, comparisons with cultured PEC that received no stimulation *in vitro* (light green bars) are indicated with ∗ and comparisons with cultured PEC received LPS (red bars) or LPS+IFN-*γ* (yellow bars) are indicated with ≠. Comparisons among the *in vivo* experimental groups are indicated with ∗∗. (e) Recombinant LieIF protein elicits the secretion of MIP-1*α* by PEC. 24 h post-immunization, PEC were harvested from each experimental group and were further incubated *in vitro* with LieIF (10 *μ*g/mL), IFN-*γ* (1 ng/mL), and LPS (1 *μ*g/mL) or with LieIF+IFN-*γ* and LPS+IFN-*γ*, for 24 h at 37°C in the presence of 5% CO_2_ environment. At the end of incubation period, culture supernatants were collected and MIP-1*α* levels were determined by ELISA. Data are presented as mean values ± SD of three independent experiments. For each *in vivo* experimental group, comparisons with cultured PEC that received no stimulation *in vitro* (light green bars) are indicated with ∗, comparisons with cultured PEC that received LPS (red bars) or LPS+IFN-*γ* (yellow bars) are indicated with ≠, and comparisons between cultured PEC that received LieIF (light blue bars) and LieIF+IFN-*γ* (orange bars) are indicated with ≠≠. Comparisons among the *in vivo* experimental groups are indicated with ∗∗.

**Figure 3:**
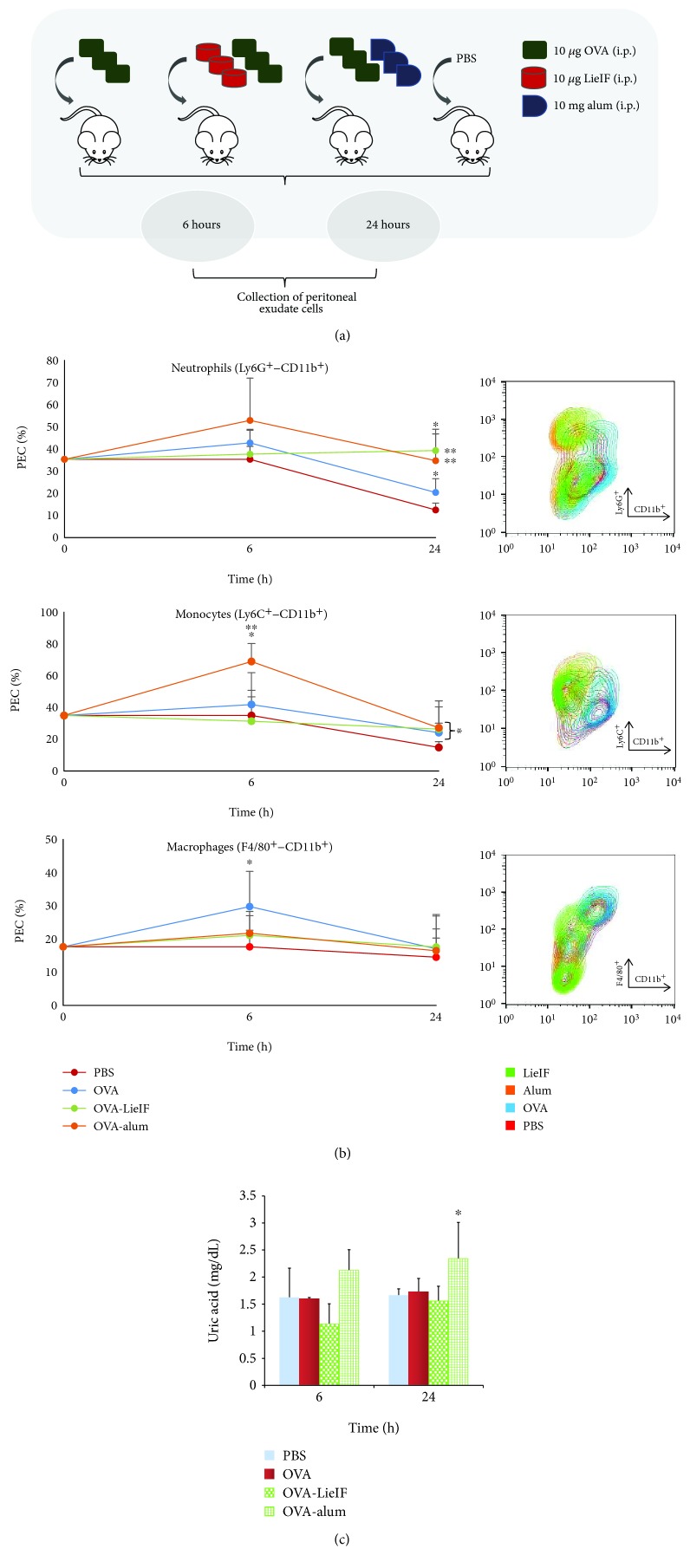
*In vivo* effect of recombinant LieIF protein on the response of innate immune cells. (a) Schematic representation of the experimental protocol. Female BALB/c mice were i.p. injected in the right quadrant with 500 *μ*L of LieIF suspension (10 *μ*g/mouse) in sterile PBS containing equal quantity of OVA protein (10 *μ*g/mouse) (OVA-LieIF) or with 500 *μ*L of OVA suspension (10 *μ*g/mouse) in sterile PBS. Mice of the positive control received the known adjuvant alum (10 mg/mouse) in combination with OVA (10 *μ*g/mouse) (OVA-alum). Mice receiving only PBS were also included. Six and 24 h after injection, the peritoneal exudate cells (PEC) were harvested with 5 mL of ice-cold PBS. (b) Recombinant LieIF protein recruits innate immune cells to the peritoneal cavity. Six and 24 h after immunization, the peritoneal lavage was harvested and the percentages of neutrophils (Ly6G^+^-CD11b^+^), monocytes (Ly6C^+^-CD11b^+^), and macrophages (F4/80^+^-CD11b^+^) were determined by FACS. Results are presented in 2D line charts and in representative contour plots. ∗ indicates statistical difference compared with the PBS-immunized mice (negative control group), and ∗∗ indicates statistical difference compared to OVA-immunized mice. (c) The immunopotentiating effect of LieIF does not depend on the production of uric acid. The levels of uric acid (mg/dL) were measured in serum of immunized mice using the enzymatic colorimetric uricase PAP method. The results are presented as the mean ± SD, and data shown are representative of three independent experiments. ∗ indicates statistically significant differences as compared to the negative control.

Female BALB/c mice, *n* = 20/group, were injected intraperitoneally (i.p.) in the right lower quadrant using a 26-gauge needle, with 500 *μ*L of LieIF suspension (10 *μ*g/mouse) in sterile PBS containing equal quantity of OVA (10 *μ*g/mouse) (OVA-LieIF), or with 10 *μ*g of OVA alone in 500 *μ*L PBS, while mice receiving only PBS were included as negative control (Figure 2(a)), as previously described [16]. In another set of experiments, BALB/c mice received the known adjuvant alum (10 mg/mouse) in combination with OVA (10 *μ*g/mouse) (OVA-alum) (Figure 3(a)). Two, 6, and 24 h after injection, the peritoneal exudate cells (PEC) were harvested with 5 mL of ice-cold PBS. Cells were depleted from red blood cells with ammonium-chloride-potassium lysing buffer (ACK buffer), pH = 7.2 (0.15 M NH_4_Cl, 10 mM KHCO_3_, and 0.1 mM Na_2_EDTA) and resuspended in complete RPMI-1640 medium.

### 2.6. Flow Cytometry

For the detection of B7 co-stimulatory molecules (CD80 and CD86) in J774A.1 macrophages, cells were stimulated with LieIF (10 *μ*g/mL) for 24 h, at 37°C with 5% CO_2_ environment. The protein concentration was carefully selected after concentration kinetic experiments [24]. As a positive control for macrophage stimulation, J774A.1 cells were cultured with LPS (1 *μ*g/mL) derived from *Escherichia coli* (Sigma-Aldrich, USA), as previously described in similar experimental conditions [30]. Accordingly, PEC were harvested in ice-cold PBS, as described in Section 2.5. At the end of the incubation period, cells were centrifuged at 600 × g for 10 min and then were resuspended in PBS at a density of 5 × 10^6^ cells/mL. Cells were washed in FACS buffer (PBS-3% FBS) and were stained with anti-CD80 and anti-CD86 monoclonal antibodies conjugated with fluorescein (FITC) (BD Biosciences, Belgium), for 30 min.

For the detection of recruited cells at 6 and 24 h after intraperitoneal injections, cell suspensions of peritoneal lavage were centrifuged at 600 × g for 10 min. The cell pellets were resuspended in PBS and stained with anti-CD11b monoclonal antibody conjugated with FITC together with anti-F4/80 monoclonal antibody conjugated with phycoerythrin (PE) (AbD Serotec, UK) or anti-Ly6C or anti-Ly6G monoclonal antibodies conjugated with PE (BD Biosciences, Belgium), for 30 min.

Control unstained samples were similarly processed for all the above cases. 20,000 events were analyzed for each sample in a FACSCalibur cytometer (Becton-Dickinson, San Jose, CA, USA), and data were analyzed with FlowJo V.10.0.8 software (Tree Star Inc., Ashland, OR, USA).

### 2.7. Chemokine Production

J774A.1 macrophages were incubated with LieIF for 24 h, at 37°C in the presence of 5% CO_2_. Macrophages cultured with LPS (1 *μ*g/mL) or cultured only with complete RPMI-1640 medium were used as positive and negative controls, respectively. Accordingly, for the determination of MIP-1*α* in PEC derived from immunized mice as described in Section 2.5, cells (at a density of 1 × 10^6^ cells/mL) were further incubated *in vitro* with the following antigens: LieIF (10 *μ*g/mL), recombinant murine (rm) IFN-*γ* (1 ng/mL), LPS (1 *μ*g/mL) [31], or with combinations of LieIF+IFN-*γ* or LPS+IFN-*γ*, for 24 h at 37°C under 5% CO_2_ environment. At the end of the incubation periods, culture supernatants were collected to determine MIP-1*α* chemokine levels by ELISA. The ELISA kit (900-K125) was purchased from PeproTech Corp. (Rocky Hill, NJ), and the assay was performed according to the manufacturer's instructions. The concentration of MIP-1*α* was calculated by using a standard curve generated by recombinant MIP-1*α* starting at 0.5 ng/mL and serially diluted in duplicate. The detection threshold was at 8 pg/mL.

### 2.8. Quantification of Extracellular Nitric Oxide (NO)

The NO synthesis was measured as the accumulation of nitrites in cell culture supernatants using the Griess reaction (Sigma-Aldrich, USA), according to manufacturer's protocol [32]. For the determination of NO in J774A.1 macrophages, cells were stimulated with LieIF for 24 h and then culture supernatants were collected. Macrophages cultured with LPS (1 *μ*g/mL) were used as the positive control and cells cultured only with complete RPMI-1640 medium constituted the negative control. Accordingly, for the determination of NO in PEC derived from immunized mice as described in Section 2.5, cells (at a density of 1 × 10^6^ cells/mL) were further incubated *in vitro* with the following antigens: LieIF (10 *μ*g/mL), IFN-*γ* (1 ng/mL), LPS (1 *μ*g/mL), or with combinations of LieIF+IFN-*γ* or LPS+IFN-*γ*, for 24 h at 37°C under 5% CO_2_ environment [33]. At the end of the incubation period, culture supernatants were collected.

50 *μ*L of each sample supernatant was mixed with 100 *μ*L of Griess reagent (1 : 1 solution A : solution B; 1% *w*/*v* sulfanilamide in 5% *w*/*v* phosphoric acid (solution A) and 0.1% *v*/*v* naphthylethylenediamine dihydrochloride in distilled water (solution B)) [34]. The relative NO concentrations were calculated using a standard curve generated with known amounts of NaNO_2_, and the absorbance was measured at 570 nm with a Dynatech Laboratories MRX spectrophotometer (Germany).

### 2.9. Gene Expression Analysis

PEC were derived from immunized and non-immunized BALB/c mice (Section 2.5) at 2 h post-immunization, and RNA was extracted using an RNeasy Mini Kit (Qiagen, Germany) according to manufacturer's instructions. The quantity and purity of extracted RNA were determined with the spectrophotometer NanoDrop® 2000 (Thermo Scientific, USA). RNA (1 *μ*g) was used as a template for cDNA synthesis using a SuperScript II kit (Invitrogen Molecular Probes™) and oligo-dTs (Promega, WI, USA), and all reactions included the recombinant ribonuclease inhibitor, RNaseOUT™ (Invitrogen).

Real-time polymerase chain reaction (real-time PCR) was performed using an Exicycler 96 (Bioneer, Daejeon, Korea) with a SYBR Green PCR Master Mix (Kapa Biosystems, Boston, USA). The expression of the g*lyceraldehyde*-*3*-*phosphate dehydrogenase* (*GAPDH*) gene was used for normalization. Specific primers for genes of interest: interleukin-1*β* (IL-1*β*), tumor necrosis factor-*α* (TNF-*α*), subunit 1 and subunit 2 of nuclear factor kappa-B (NF-*κ*B1 and NF-*κ*B2), and *GAPDH* were designed by Qiagen (QuantiTect Primer Assays; Qiagen, Netherlands) and were run in triplicate. The PCR was conducted according to Qiagen's PCR protocol for the QuantiTect Primer Assays. The cycling conditions were 94°C for 10 min, followed by 40 cycles at 94°C for 10 s and 60°C for 30 s. All expression levels were computed via the ΔΔCt method [35].

### 2.10. Serum Uric Acid Determination

Serum samples were collected from immunized mice, described in Section 2.5, at 6 and 24 h post-immunization. Serum uric acid (SUA) levels in (mg/dL) were determined by the enzymatic colorimetric uricase PAP method [36], according to manufacturer's instructions and using a Cobas Mira autoanalyzer (Roche, Switzerland), kindly accessed by V. Sideris, MD, at Diagnostiki Athinon, Clinical and Research Laboratory (Athens, Greece).

### 2.11. Statistical Analysis

The data shown are representative of at least three independent experiments and are presented as mean values ± standard deviation (SD). In the *in vivo* procedures, we used six to seven animals per group and the experiments were repeated three times. Statistical analysis was performed by the two-sided Mann-Whitney test using the IBM SPSS Statistics software (v.24). *P* values less than 0.05 were considered to indicate statistical significance.

## 3. Results

### 3.1. Recombinant LieIF Induces the Upregulation of CD80 and CD86 Co-stimulatory Molecules in J774A.1 Macrophages

**Figure 4:**
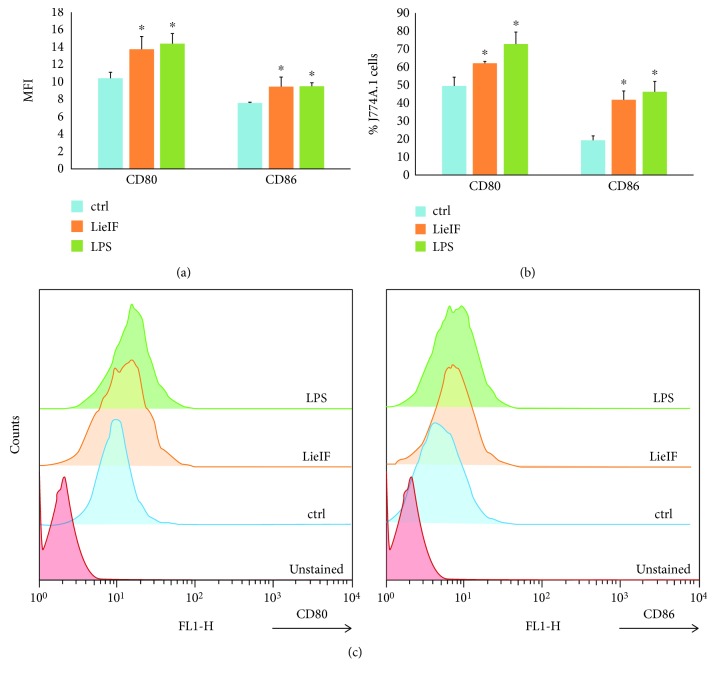
Effect of recombinant LieIF protein on the expression of co-stimulatory molecules by J774A.1 macrophages. Macrophages were stimulated with recombinant LieIF (10 *μ*g/mL) for 24 h, and the expression of CD80 and CD86 molecules was measured using FACS with the use of specific monoclonal fluorochrome-labeled antibodies. Macrophages stimulated with LPS (1 *μ*g/mL) were used as the positive control while unstimulated cells were used as the negative control. The results are expressed as (a) median fluorescent intensity (MFI) and (b) percentage (%) of macrophages expressing CD80 and CD86 molecules. Data are presented as mean values ± SD of three independent experiments. (c) Histogram overlays are representative of one experiment. ∗ indicates statistically significant differences as compared to the negative control.

LieIF was expressed and purified by Ni-affinity chromatography and its purity was more than 90% (Figure 1). Firstly, the phenotypic changes of murine macrophages in response to LieIF were analyzed; since the expression of co-stimulatory molecules (e.g., CD80, CD86) on APCs, macrophages, and DCs is critical in shaping the extent and nature of immune responses [37]. LieIF-stimulated macrophages were labeled with antibodies directed against the B7 surface markers (CD80 and CD86). FACS analysis showed that stimulation of J774A.1 macrophages with LieIF induced a significant increase in the expression of CD80 and CD86 co-stimulatory molecules in terms of MFI (Figures 4(a) and 4(c)) along with the percentage (%) of J774A.1 cells (Figures 4(b) and 4(c)). Specifically, LieIF-stimulated macrophages exhibited a 1.3-fold increase of MFI of cells expressing both CD80 and CD86 molecules (Figures 4(a) and 4(c)) along with a 1.3- and a 2.2-fold increase of % of J774A.1 cells expressing CD80 (62.1 ± 2.5% vs. 49.4 ± 4.8%) and CD86 (41.8 ± 4.8% vs. 19.2 ± 1.05%), respectively, as compared with unstimulated cells (Figures 4(b) and 4(c)). It is also noteworthy that LieIF-stimulated macrophages exhibited similar expression of CD80 and CD86 molecules as compared to the expression caused by LPS-stimulated cells (*p* = 0.386 and 0.657, respectively) (Figure 4).

### 3.2. Recombinant LieIF Induces the Production of Nitric Oxide and MIP-1*α* Chemokine by J774A.1 Macrophages

**Figure 5:**
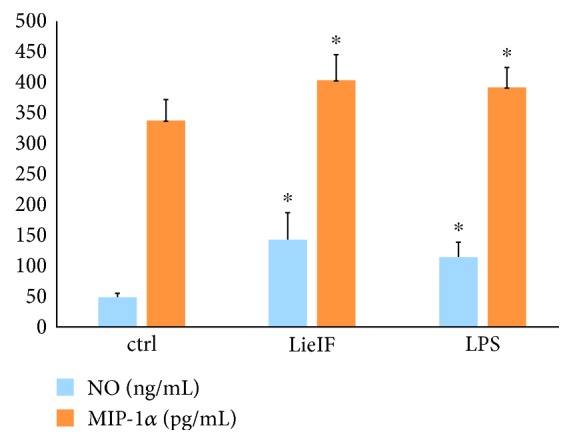
Effect of recombinant LieIF protein on nitric oxide (ng/mL) and MIP-1*α* (pg/mL) production by J774A.1 macrophages. Macrophages were stimulated with recombinant LieIF (10 *μ*g/mL) and LPS (1 *μ*g/mL). Unstimulated cells were used as the negative control. After 24 h, the cell supernatants were collected and NO and MIP-1*α* secretion were measured by Griess reaction and ELISA, respectively. The results are presented as the mean ± SD and data shown are representative of three independent experiments. ∗ indicates statistically significant differences as compared to the negative control.

NO production is a marker for macrophage activation and one of the major antimicrobial mechanisms of macrophages. Indeed, sustained production of NO endows macrophages with cytotoxic activity against viruses, bacteria, fungi, protozoa, helminths, and tumor cells [38]. NO levels were measured in culture supernatants of J774A.1 macrophages after their *in vitro* stimulation with LieIF and the amount of the released NO is given in Figure 5. The obtained data indicated that NO production in LieIF-stimulated cells was significantly higher (141.89 ± 44.63 ng/mL) as compared to unstimulated cells (47.77 ± 6.9 ng/mL, *p* = 0.004) and the amount of produced NO was almost equal to the levels produced by the LPS-stimulated J774A.1 cells (113.15 ± 25.33 ng/mL, *p* = 0.327) (Figure 5).

On the other hand, chemokines play an important role in the selective movement of leucocytes into areas of inflammation [39]. Macrophage inflammatory protein-1 alpha (MIP-1*α*) is a member of the CC chemokine family and is a chemotactic attractant for lymphocytes, monocytes, and eosinophils [40]. MIP-1*α* levels were also measured in culture supernatants of J774A.1 macrophages after their *in vitro* stimulation with LieIF, and the amount of the produced MIP-1*α* is also shown in Figure 5. Clearly, LieIF induced the secretion of statistically significant amounts of MIP-1*α* chemokine by J774A.1 macrophages *in vitro* as compared to unstimulated cells (402.76 ± 42.6 pg/mL vs. 337.15 ± 34.5 pg/mL, *p* = 0.045) while these amounts were similar to those produced by LPS-stimulated J774A.1 macrophages (391.47 ± 32.78 pg/mL, *p* = 0.855).

### 3.3. Effect of Recombinant LieIF Co-administered with OVA Antigen on the Innate Immune Response Elicited after Intraperitoneal Injection

BALB/c mice were intraperitoneally immunized either with OVA antigen alone or with LieIF protein together with OVA antigen dissolved in PBS, or PBS alone as negative control, as it is shown in Figure 2(a). At 2 and 24 h after injection, PEC were harvested in order to determine the primary response induced. The expression of proinflammatory immune genes and immune mediators that may indicate the capacity of LieIF to trigger locally *in vivo* an immunological profile describing an adjuvant activity was measured.

#### 3.3.1. Recombinant LieIF Induces a Proinflammatory Environment at the Injection Site

Most of the times, adjuvants are associated with the transient secretion of cytokines and chemokines which mediates the formation of a local proinflammatory environment composed of various immune cells recruited to the injection site [41]. Production of IL-1*β*, IL-6, and TNF-*α* cytokines is one of the hallmarks of the inflammatory response and plays an important role in the initiation of innate immunity [42, 43]. The relative expression of *TNF-α*, *IL-1β*, and *NF-κB2* genes was determined by real-time PCR at 2 h post-immunization. Obtained data demonstrated that co-administration of OVA-LieIF is able to induce a proinflammatory environment at the injection site as illustrated by the elevated gene expressions. More specifically, *TNF-α* gene expression in PEC from OVA-LieIF-immunized mice was 25.8- and 1.6-fold upregulated compared to the corresponding expression level in PEC from PBS- and OVA-immunized mice (*p* = 0.037 and 0.456, respectively; Figure 2(b)). Furthermore, the *IL-1β* gene expression in mice that received OVA-LieIF was 585- and 61.5-fold upregulated as compared to that in PBS- and OVA-immunized mice (*p* ≤ 0.050; Figure 2(b)). At last, we determined the expression of two members of the NF-*κ*B family *NF-κB1* and *NF-κB2* genes that play an important role in the regulation of immune and inflammatory responses. We observed an upregulation of 648- and 40.6-fold of *NF-κB2* gene expression in PEC from OVA-LieIF-immunized mice versus the control groups of PBS- and OVA-immunized mice, respectively (*p* ≤ 0.050; Figure 2(b)). The *NF-κB1* gene expression was equal in mice of both immunized groups (data not shown).

#### 3.3.2. Effect of Recombinant LieIF on the Functional Maturation of PEC

At first, changes in the expression of co-stimulatory molecules in PEC, elicited by OVA-LieIF intraperitoneal co-administration, were analyzed and it was found that PEC derived from OVA-LieIF-immunized mice exhibited elevated expression of CD80 molecule in terms of MFI along with the percentage (%) of cells (Figure 2(c)). Specifically, PEC from OVA-LieIF-immunized mice exhibited a 1.3-fold increase of MFI as compared with both PBS- and OVA-immunized mice (*p* ≤ 0.050) at 24 h post-injection. Moreover, OVA-LieIF-immunized mice exhibited an elevated number of cells expressing CD80 (24.6 ± 4.9%) as compared with PBS- (17.1 ± 2.4%) and OVA- (16.9 ± 0.4%) immunized mice (*p* = 0.023 and 0.004, respectively), at 24 h post-injection (Figure 2(c)). No upregulated expression of CD86 was induced by OVA-LieIF or OVA administration (Figure 2(c)).

Moreover, the NO levels in culture supernatants of PEC obtained from immunized mice of each experimental group were also determined. *In vitro* restimulation of PEC with LieIF alone or LieIF+IFN-*γ* led to increased NO production in all experimental groups as compared with the NO produced from unstimulated cells (*p* values ranging from 0.009 to 0.034) (Figure 2(d)). Likewise, stimulation with LieIF+IFN-*γ* resulted also in equal or higher amounts of NO production in all experimental groups, as stimulation with LPS or LPS+IFN-*γ*, respectively (recorded *p* values 0.046, 0.050, 0.275, and 0.289) (Figure 2(d)). Unstimulated cells derived from OVA-LieIF-immunized mice demonstrated a moderate increase of NO production when compared to the PBS- or OVA-immunized mice (*p* = 0.091). Of note, the immunization with OVA-LieIF promoted noticeably increased NO levels upon stimulation with LieIF or LieIF+IFN-*γ* as compared with PBS- and OVA-immunized mice also restimulated with LieIF±IFN-*γ* (*p* values 0.046 and 0.009) (Figure 2(d)). Overall, the data on NO production indicated that LieIF promoted macrophage activation when administered *in vivo*.

Next, it was determined whether coadministration of OVA-LieIF elicited the production of MIP-1*α*, the known chemotactic attractant for lymphocytes. MIP-1*α* was measured in PEC culture supernatants after *in vitro* restimulation with LieIF±IFN-*γ* and LPS±IFN-*γ* for 24 h. The obtained data showed that the secreted levels of MIP-1*α* were significantly higher in supernatants of PEC restimulated *in vitro* with recombinant LieIF±IFN-*γ* than those of unstimulated cells (*p* values ranging from 0.009 to 0.018), for all the experimental groups (Figure 2(e)). It was also noticed that stimulation with LieIF+IFN-*γ* resulted in equal or higher levels of secreted MIP-1*α* in all experimental groups, as compared to those induced by stimulation with LPS or LPS+IFN-*γ*, respectively (recorded *p* values 0.018, 0.237, and 1.000) (Figure 2(e)). It is noteworthy that the induction of MIP-1*α* secretion was significantly enhanced in unstimulated PEC derived from OVA-LieIF-immunized mice than that of corresponding secretion from the PBS- or OVA-immunized mice (*p* = 0.018). Moreover, OVA-LieIF-immunized mice had an enhanced MIP-1*α* production upon stimulation with LieIF±IFN-*γ* (Figure 2(e)), as compared to PBS- and OVA-immunized mice (recorded *p* values 0.009, 0.083, and 0.237).

### 3.4. Assessment of the Effect of Recombinant LieIF on the Response of Innate Immune Cells

BALB/c mice were either immunized with LieIF protein together with an equal amount of OVA antigen (OVA-LieIF), or with alum plus OVA (OVA-alum), while other mice received OVA antigen alone. Mice of the negative control group received sterile PBS (Figure 3(a)). The innate immune response to LieIF in the peritoneum, 6 and 24 h after immunization, was investigated.

Within 24 h after injection, OVA-LieIF administration induced a marked increase of Ly6G^+^-CD11b^+^ neutrophils, as compared to PBS- and OVA- immunized mice (*p* = 0.034 and 0.025, respectively; Figure 3(b)). The effect of LieIF on the recruitment of neutrophils was similar to alum (*p* = 0.456; Figure 3(b)). Furthermore, at the same time point, as compared to PBS-immunized mice, OVA-LieIF led to a significant recruitment of inflammatory Ly6C^+^-CD11b^+^ monocytes (*p* = 0.023; Figure 3(b)), previously shown to be immediate precursors for DCs [44, 45]. As it is shown here, LieIF leads to the recruitment of cells that coincide to key players of the inflammatory reaction such as neutrophils and monocytes. The results reported here revealed that the alterations in peritoneal cell populations elicited by co-administration of OVA-LieIF almost mimicked the alterations elicited by alum.

It has been demonstrated that alum induces a strong neutrophilic influx accompanied by the production of IL-1*β*, akin to the response seen when the endogenous danger signal, uric acid, is injected into the peritoneal cavity [46, 47]. Kool et al. demonstrated that the immunopotentiating effect of alum depends on the induction of uric acid. In the present study, we checked if the immunopotentiating effect described above and attributed to LieIF was mediated by the presence of uric acid acting as a danger signal. Our results indicated that the administration of LieIF did not reveal an increase in uric acid levels (Figure 3(c)), suggesting the induction of a different mechanism of attraction of cells to the peritoneal cavity as compared to that induced by alum.

## 4. Discussion

Only a handful of adjuvants are approved for prophylactic vaccination of humans, despite their obvious benefits, decades of research, and hundreds of preclinical candidates [11]. Failures of adjuvants during the development phase are related to the manufacturing process (e.g., lack of a reproducible formulation, negative impact on antigen stability) or to local or systemic adverse events [12]. Recent advances in the immune pathways involved in the modulation of the host-protective immune response have opened new avenues to design improved vaccine adjuvants [12].

In this study, we have demonstrated that the recombinant *Leishmania infantum* eukaryotic initiation factor (LieIF) acts as a prostimulatory agent on monocytic cell types in *in vitro* and *in vivo* assays by inducing innate immune responses and could be considered as a potential molecular adjuvant. Initially, LeIF had been proven as a potent inducer of immunity exhibiting advantageous immunomodulatory properties such as the induction of production of IL-12, IL-10, and TNF-*α* by monocytes, macrophages, and DCs derived from healthy volunteers [20, 22, 23]. Moreover, we have recently demonstrated using reverse vaccinology approaches that selected parts of LieIF can be used to develop innovative subunit protective vaccine candidates able to induce effective immunity mediated by MHC class I-restricted as well as class II-restricted T cell responses [48, 49]. Additionally, LeIF has been harnessed as a vaccine adjuvant targeted to cancer [29]. Collectively, these data suggested the potential of LeIF as a vaccine adjuvant that deserves further investigation. Our study features a number of important strengths towards the assessment of its adjuvant properties in *in vitro* and *in vivo* assays. To our knowledge, this is the first study that evaluated the ability of LieIF protein to induce the upregulation of both CD80 and CD86 macrophage surface molecules which is indispensable for the activation of T cells by APCs [50]. Also, we have demonstrated that LieIF induced increased expression of co-stimulatory molecules CD86, CD80, and CD40 in murine BMDCs (unpublished data), in consistence with another study where *L. braziliensis* eIF protein (LbeIF), having 98% identity with LieIF, was reported to induce upregulation of CD80 on human monocyte-derived macrophages [20]. Moreover, in the present study, we explored the ability of LieIF to activate host macrophages as demonstrated by the production of NO and MIP-1*α* chemokine in the supernatants of LieIF-stimulated J774A.1 macrophages [22]. These immune mediators are regulators of inflammatory responses since NO is an effector molecule in macrophage-mediated cytotoxicity [51] and MIP-1*α* is a chemoattractant mediator to a variety of cells including monocytes, eosinophils, and T and B cells to sites of infection, leading to the clearance of the microorganisms [52].

The introduction of an adjuvant in new vaccine formulation or in already licensed vaccine is still a challenge and may take several years of intensive research [15]. Thus, the understanding of their mechanism(s) of action would facilitate the acceleration of the development of effective adjuvants. In this regard, we further evaluated the potential of LieIF to provide adjuvant properties in an *in vivo* murine experimental model. Although, as mentioned above, some reports have indicated that LeIF is a natural Th1-type adjuvant [21], this is the first study documenting the adjuvant properties of LieIF using a murine model. In the present study, noticeable side effects such as abnormal behavior were not observed after intraperitoneal administration of LieIF. Analysis of immune parameters, such as phenotypic and functional differentiation of the cells locally recruited in the peritoneum after LieIF administration, revealed that LieIF protein is able to confer adjuvant properties to OVA, a soluble protein antigen, when both were intraperitoneally administered, as illustrated by the upregulated expression of the CD80 molecule and the increased production of NO and MIP-1*α* in PEC. Macrophage activation induced by the immunization of mice with OVA-LieIF was demonstrated by induction of NO synthesis in response to *in vitro* restimulation with recombinant LieIF protein. Immunization with OVA antigen alone did not enhance NO production by PEC even after stimulation with LPS or LPS+IFN-*γ*. Furthermore, a significant MIP-1*α* release was observed in culture supernatants of PEC derived from OVA-LieIF-immunized mice compared to other experimental groups after different potent *in vitro* stimulations. Moreover, a number of observations support that a cluster of genes encoding cytokines, innate immune receptors, interferon-induced genes, and gene encoding adhesion molecules are defined as “adjuvant core response genes” since they have been found to be modulated by adjuvants such as alum, MF59, and CpG-ODN at the injection site [53]. To this end, we determined the relative expression of two prototypic proinflammatory cytokines, IL-1*β* and TNF-*α*, and demonstrated that LieIF induced significant higher levels of *IL-1β* and *TNF-α* gene expression in PEC as early as 2 h post-immunization. Collectively, the above data demonstrate that proinflammatory signals elicited by LieIF result to a proinflammatory environment at the injection site and this is indeed a mechanism common to various known adjuvants [41].

To more critically address the adjuvant properties of LieIF, we used a similar approach which had been used for alum, a widely used adjuvant in humans [16]. Mice were immunized with LieIF in combination with the poorly immunogenic OVA antigen in order to assess the cellular recruitment into the peritoneal lavage fluid. We found that LieIF was effective to provoke similar recruitment of immune cells at the injection site as compared to alum, namely, increased frequencies of neutrophils and monocytes at 24 h post-immunization, even though, in contrast with alum, the immunopotentiating effect of LieIF was not mediated by uric acid danger signal.

In conclusion, the present study provides evidence that LieIF acts as an immune potentiator by inducing a proinflammatory environment at the injection site that enables the recruitment of innate immune cells, induces cytokine expression, activates macrophages, and exhibits stimulatory effects for antigen presentation.

## 5. Conclusions

In this study, we demonstrate the adjuvant properties of LieIF that collectively suggest its potential use for novel vaccine formulations. LieIF induces the upregulation of CD80 and CD86 co-stimulatory molecules, as well as the production of the NO and MIP-1*α* immune mediators *in vitro*, by J774A.1 macrophages. Moreover, LieIF is able to promote macrophage activation and to induce a proinflammatory environment at the injection site after its intraperitoneal co-administration with OVA antigen in a murine model. Additionally, LieIF leads to the recruitment of neutrophils and monocytes at the injection site, similar to alum's effect.

## Data Availability

All data related to this study have been provided within the manuscript and are also available from the corresponding author based on a reasonable request.

## Abbreviations

APCs: Antigen-presenting cells
DCs: Dendritic cells
FACS: Fluorescence-activated cell sorter
IFN-*γ*: Interferon gamma
IL-10: Interleukin 10
IL-12: Interleukin 12
LieIF: *Leishmania infantum* eukaryotic initiation factor
LPS: Lipopolysaccharide
MIP-1*α*: Macrophage inflammatory protein-1 alpha
NO: Nitric oxide
PEC: Peritoneal exudate cells
r: Recombinant
TNF-*α*: Tumor necrosis factor alpha.
